# The emergence of spiraling tracheary element bundles in incompatible grafts

**DOI:** 10.7717/peerj.14020

**Published:** 2022-09-14

**Authors:** Huiyan Wu, Zhuying Deng, Xue Wang, Dacheng Liang

**Affiliations:** School of Agriculture, Yangtze University, Jingzhou, Hubei, China; Engineering Research Center of Ecology and Agricultural Use of Wetland, Ministry of Education, Yangtze University, Jingzhou, Hubei Province, China

**Keywords:** Interfamilial graft, Xylem, Circular vessels, Spiraling tracheary element, Incompatibility, *Portulaca oleracea*, *Brassica napus*, *Nicotiana benthamiana*, *Arabidopsis thaliana*

## Abstract

In distantly-related plant grafting, incompatibility often occurs between scion and rootstock, resulting in growth stagnation, and eventually graft failure. In this study, we found that an emergent structure, or the spiraling tracheary element (TE) bundles consisting of TE masses occurring at the graft interface, was extensively present in the highly incompatible interfamilial graft of *Brassica napus/Portulaca oleracea* (*Bn/Po*) and *Nicotiana benthamiana*/*Portulaca oleracea* (*Nb*/*Po*). This special structure mostly appeared in the local area near the grafting union, and the frequency and quantity of the spiraling tracheary element bundles were much higher in the scion than in the rootstock. Nevertheless, only a small portion of *Arabidopsis thaliana*/*Portulaca oleracea* (*At/Po*) interfamilial grafts showed a less spiraled TEs at the grafting union (usually a circular TE), which is consistent with its growth performance. This study consolidated that spiraling TE bundles were an important indicator for graft incompatibility. The possible reason for the formation of spiraling TE bundles in interfamilial grafts was discussed.

## Introduction

Grafting is one of the artificial propagation methods of plants in which two different plant segments are mechanically joined together to survive as a new individual after vascular connection and wound healing. This technique was practiced in ancient Greece and China as early as the 5th century BC (Melnyk & Meyerowitz, 2015). At present, it is widely used in agricultural production and horticultural research.

The compatibility between scion and rootstock is the key factor affecting the grafting success. A prerequisite for successful grafting and long-term survival is the taxonomic closeness of the scion to the stock. In general, different species of plants in the same genus can form effective union and survive, while grafting of plants in different genera of the same family are rarely compatible (Goldschmidt, 2014). In some special cases of grafting between distant species, such as *Arabidopsis* and tomato, despite the lack of vascular connection, the *Arabidopsis* scion grafted onto tomato rootstock can still blossom and produce seeds (Flaishman et al., 2008). However, due to the low efficiency of nutrition and hormone transfer between tissues, it is still doubtful whether these grafts can be maintained for a long time (Melnyk, 2017). So far, the mechanism of the incompatibility of heterograft is still unclear, and practical graft work relies largely on empirical observation for horticultural production.

The connection of vascular bundle after grafting is one of the important signs of grafting success (Melnyk, 2017; Melnyk & Meyerowitz, 2015). The cuts introduced during grafting necessitate the destruction of the vascular system of plants, resulting in the discontinuity of vascular transport of water, nutrients and various organic materials (Asahina & Satoh, 2015). Therefore, the connection of the vascular system between graft partners needs to be quickly re-established for survival. However, various factors including the taxonomic relatedness, anatomical structure (*e.g.*, the necrotic layer at the graft interface), mechanical mismatch during grafting, pathological infections, growth condition and growth activity of either graft partner could cause the graft failure (Hartmann et al., 2011). Mosse (1962) proposed that destruction of vascular continuity due to abnormalities of vascular development at the graft union caused graft incompatibility. In a recent study, formation of a spiraling tracheary element bundle (spiraling TE bundle) in the graft union was strongly associated with graft incompatibility in the *Arabidopsis*/*Nicotiana* interfamilial graft (Deng et al., 2021). This further suggests that vascular behavior was critical for graft union formation.

To further test whether this particular vascular structure was occurring in other interfamilial grafts, we focused on interfamilial combination involving purslane as rootstock. The existence of spiraling TEs in the interfamilial grafts, *i.e.,* the *Brassica napus/Portulaca oleracea* (*Bn/Po*), *Nicotiana benthamiana*/*Portulaca oleracea* (*Nb*/*Po*) and *Arabidopsis thaliana*/*Portulaca oleracea* (*At/Po*) combination was described, and the causes of their formation and their influence on the growth of distant grafting were also discussed.

## Material and Methods

### Experimental materials

The seeds of rape (*Brassica napus*), *Nicotiana benthamiana*, *Arabidopsis thaliana* and purslane (*Portulaca oleracea*) were surface-sterilized in chlorine gas for 2 h. Then they were plated on the sterile MS medium containing 3% sucrose (W/V). Sterile petri dishes containing seeds were vertically placed in the growth room for constant temperature growth (22∼23 °C), and the growth condition was set at long-day photoperiod cycle (16 h of light and 8 h of darkness).

### Micrografting

Seedlings germinated after 7-9 days on MS medium were selected for grafting (purslane as rootstock, rape, *Nicotiana benthamiana* and *Arabidopsis* as scion, respectively). The grafting process was described previously (Deng et al., 2021). Grafts were grown on moisturized Whatman paper for 3 days, then gently moved to MS medium containing 3% sucrose (w/v) with forceps and continued to grow in a growth room (16 h light /8 h darkness) at 22−23 °C.

### Vasculature isolation, scanning electron microscopy and counting of the spiraling TE bundles

Isolation of vasculature was recently described by Liu et al. (2022) and Deng et al. (2021). The leaves and roots of grafts were removed and soaked in 0.04% saponin solution for 30 min, then washed with PEM solution (50 mM PIPES, 5 mM EGTA, 2.5 mM MgSO4, pH 6.9) three times, and then fixed in 4% paraformaldehyde in PEM buffer for 30 min. The fixed samples were washed with PEM solution and then dissected to remove the tissues adjacent to the vascular bundles under a dissecting microscope. The longitudinally dissected vascular samples were treated with the enzyme cocktail solution consisting of 5 mM 2-(N-morpholino) ethanesulfonic acid (MES), 0.5% (w/v) cellulase (EC 3.2.1.4; Sigma-Aldrich, St. Louis, MO, USA), 0.2% (v/v) pectinase (EC 3.2.1.15; Sigma-Aldrich, St. Louis, MO, USA), 0.12 M sucrose, 1 mM CaCl_2_(pH 5.5) at 28 °C for 1.5 h. The treated samples were washed in 5% TritonX-100 for 15 min and then in PEM solution for 15 min. Samples were dehydrated step by step (15 min each) with 15%, 30%, 50%, 75% and 100% ethanol. With another two times of absolute ethanol wash, they were dried for 3 h in a −20 °C, using a low-vacuum drier (CHRIST). The dried samples were mounted on the sample holder, and then placed in the ion sputtering apparatus (SC7620 Sputter coater) for conductive gold coating. Examination of the samples was conducted in a VEGA3 TESCAN scanning electron microscope at 20 kv acceleration voltage. In order to count for the number of spiraling bundles, we surveyed the area within a three mm radius centered at the midpoint of the grafting interface. The spiraling bundle was characterized with spiraling tracheary elements plus either a concave hole or convex point, and thus was counted as a spiraling bundle. The *Arabidopsis*/purslane grafts (*n* = 67) were surveyed every two days after grafting (DAG), while rape/purslane grafts (*n* = 35) and *Nicotiana*/purslane grafts (*n* = 43) were mainly surveyed after 28 DAG. For each time of collection, graft unions from at least six individual grafts were prepared.

## Results

### Three interfamilial grafts using purslane as rootstock

**Figure 1:**
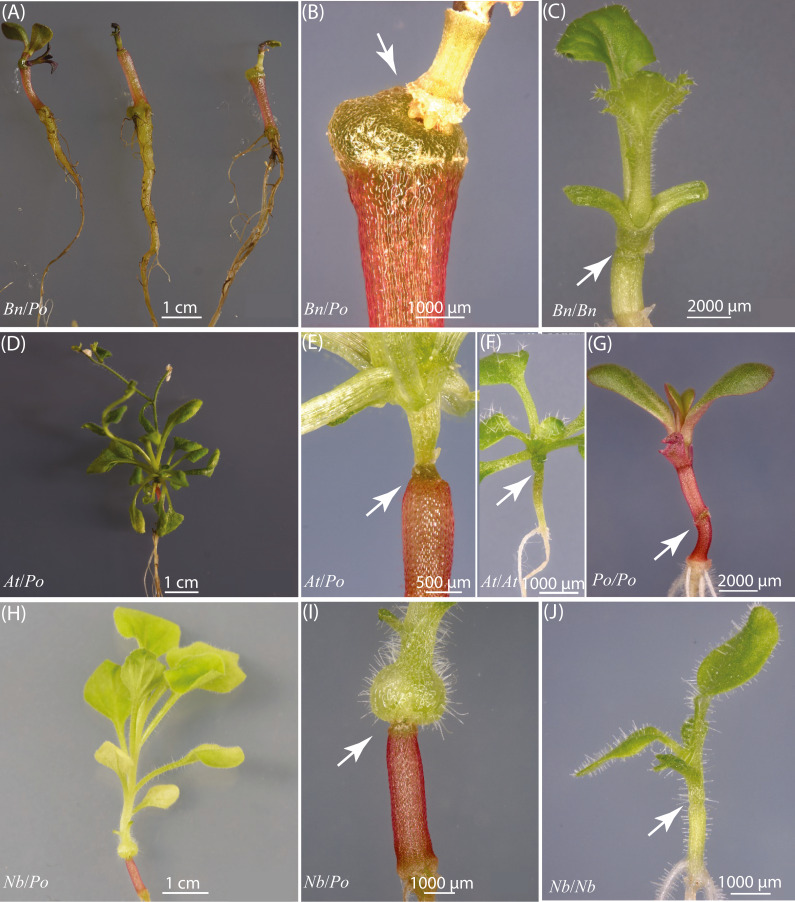
Heterografts of *Bn*/*Po*, *At*/*Po* and *Nb*/*Po* combination and self-grafts of *Bn*, *At*, *Po* and *Nb* species. (A) The representative plants of *Bn*/*Po* heterografts showing growth stagnation. (B) The graft union of the *Bn*/*Po* heterograft. (C) *Bn* self-graft. (D) A representative plant of *At*/*Po* heterograft. (E) The graft union of the *At*/*Po* heterograft. (F) *At* self-graft. (G) *Po* self-graft. (H) A representative plant of *Nb*/*Po* heterograft. (I) The graft union of the *Nb*/*Po* heterograft. (J) *Nb* self-graft. Arrow indicates the graft union.

In this experiment, we constructed three interfamilial grafts, the *Bn*/*Po* heterograft (Figs. 1A and 1B), *At*/*Po* heterograft (Figs. 1D and 1E), and *Nb*/*Po* heterograft (Figs. 1H and 1I), and four self-grafts, *i.e.,* the *Bn* (Fig. 1C), *At* (Fig. 1F), *Po* (Fig. 1G) and *Nb* (Fig. 1J) self-graft by micrografting technique. Nearly all the *Bn*/*Po* grafts remained quiescent (Fig. 1A), a growth status similar to some grafts of *At*/*Nb* combination described previously (Deng et al., 2021), suggesting the two species were highly incompatible. The *At*/*Po* grafts, however, grew very quickly at 20 DAG, suggesting that *Arabidopsis* and purslane was potentially compatible with each other at this stage (Fig. 1D). Usually, a delayed incompatibility was observed around 30 DAG; either the grafts ceased growth, or the grafts developed adventitious roots at the graft union (Table 1). The *Nb*/*Po* grafts displayed yellowing of foliage (Fig. 1H, Table 1), suggesting poor development of scion shoot. In addition, the graft unions in both *Bn*/*Po* and *Nb*/*Po* grafts were enlarged (Figs. 1B and 1I), implying a structural expansion within the unions. In the compatible self-grafts of *Bn*, *At*, *Po* and *Nb*, the growth of each species was normal (Figs. 1C, 1F, 1G and 1J).

**Table 1:** Graft compatibility and occurrence of spiraling bundles.

Grafts	Graft union	Adventitious roots	Retarded growth	Yellowing of foliage	Occurrence of Spiraling bundles	Graft Compatibility
*Bn*/*Po*	Enlarged	17.1%	82.9%	51.4%	80%	Incompatible
*At*/*Po*	Smooth	58.7%	17.4%	54.3%	16.3%	Partially incompatible
*Nb*/*Po*	Enlarged	60.7%	16.6%	100%	64.2%	Highly incompatible
*At*/*At*	Smooth	1%	0	0	0	Fully compatible
*Po*/*Po*	Smooth	0	0	0	0	Fully compatible
*Nb*/*Nb*	Smooth	0	0	0	0	Fully compatible

### Emergence of spiraling TE bundles or circular TE at the graft union

**Figure 2:**
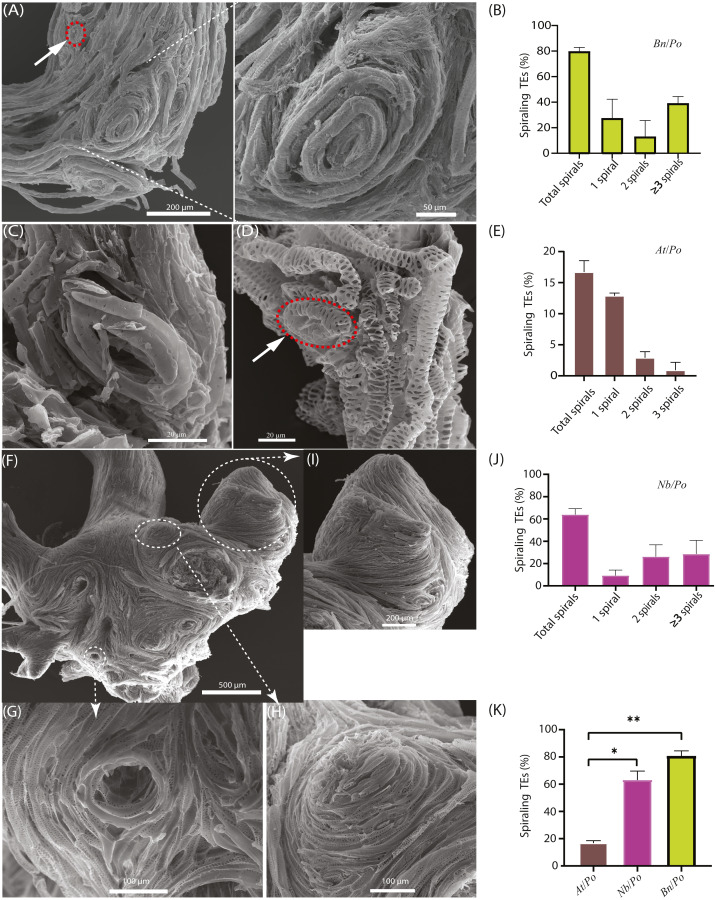
Occurrence of spiraling TE bundles in interfamilial graft. (A) A typical *Bn*/*Po* graft interface showing the widespread formation of spiraling TE bundles in the scion of *Bn*/*Po* graft at 28 DAG. The red circle indicates a single circular TE of rape. (B) Occurrence of various spirals in *Bn*/*Po* grafts. Data (mean ± SD) were generated in three repeats (*n* = 9, 12, and 14 respectively). (C) A small circular TE occurring in the scion of *At*/*Po* graft at 15 DAG. (D) A small circular TE (the red circle) occurring in the rootstock of *At*/*Po* graft at 27 DAG. (E) Occurrence of various spirals in *At*/*Po* grafts. Data (mean ± SD) were generated in three repeats (*n* = 20, 22, and 25 respectively). (F) The widespread formation of spiraling TE bundles in the scion of *Nb*/*Po* graft at 28 DAG. (G) The concave spiral. (H) The convex spiral. (I) The fused spirals were formed into chignon-like sphere structure. (J) Occurrence of various spirals in *Nb*/*Po* grafts. Data (mean ± SD) were generated in three repeats (*n* = 10, 13, and 20 respectively). (K) Occurrence percentage (mean ± SD) of spiraling TEs between *At*/*Po*, *Nb*/*Po* and *Bn*/*Po* grafts. Student’s *T*-test was used to generate the *p*-value. An asterisk (*) and two asterisks (**) indicated *p* < 0.05 and *p* < 0.01, respectively.

The scion and the rootstock of the *Bn*/*Po* heterografts were easily parted during the preparation for SEM, implying fragile connection between scion and rootstock. The SEM examination showed that spiraling TE bundles extensively existed at the graft union of *Bn*/*Po* combination (Fig. 2A), and around 80% of grafts produced spiraling TE bundles at the graft union (Fig. 2B). These spiraling bundles were mainly confined to the upper part of grafting interface, and they strongly rejected the connection with TEs from rootstock. A single circular tracheary element could also be formed through self-fusion as shown in Fig. 2A (shown in red circle), and a repeated process of circling led to the formation of spiraling bundles (Fig. 2A). Apparently, these spiraling bundles lost their TE end, and did not provide a tapered or inclined end wall for overlapping or fusion as normal TE did, therefore could not make a connection with the TEs from rootstock.

In *At*/*Po* grafts, the frequency of spiraling TE bundle formation was relatively low (Figs. 2C and 2D), and about 16.7% of grafts (10 out of 60) harbored circular TEs (Fig. 2E). Moreover, the TEs were loosely spiraled (Fig. 2C), thus they usually existed as a single ring and rarely formed a highly spiraling structure (Figs. 2C and 2D). In fact, the *At*/*Po* grafts grew well in the first 20 days, agreeing with the less-forming spiraling bundles.

We further examined the spiraling TEs in the scion of *Nicotiana benthamiana* belonging to solanaceae family, diverging more than 100 million years ago from brassicaceae family according to Ku et al. (2000). Similarly, the enlarged union was consisted of more spiraling TEs (Fig. 2F) which were either convolute concave (Fig. 2G) or convex (Fig. 2H). In an extreme case, these small spirals were fused to form chignon-like structure (Fig. 2I). More than 65% of these grafts contained various numbers of spirals, of whom the majority were two spirals (Fig. 2J). In all compatible self-grafts, there was no spiraling TE bundle detected (Table 1). Taken together, the more compatible *At*/*Po* combination has less spiraling TEs than in *Bn*/*Po* and *Nb*/*Po* combination (Fig. 2K, Table 1), suggesting a close association of graft incompatibility with emergence of spiraling TEs.

## Discussion

In our recent study, we found that the spiraling TE bundles existed in almost all the quiescent grafts of *Arabidopsis/Nicotiana* combination, but they appeared rarely in the grafts with active growth (Deng et al., 2021). These bundles were most likely built on circular vessels (Deng et al., 2021). The circular vessels were observed in injury-induced wood tumor of *Picea excelsa* (Lam.) Lk (Włoch, 1976). Similarly, the circular vessels were also formed close to transverse wounds, *e.g.*, the wounds at the inflorescence stems of *Arabidopsis* (Mazur, Benková & Friml, 2016), at the basal side of the radish root and in basal swellings above the cut surfaces of pea stems about a week after the plant was cut (Sachs, 1981; Sachs & Cohen, 1982). They were also formed at branch junctions of various tree species (Lev-Yadun & Aloni, 1990; Rothwell & Lev-Yadun, 2005), in the suppressed or dormant buds that were oriented across the trunk of *Ficus religiosa* (Aloni & Wolf, 1984). Usually, these circular structures occurred relatively rare and irregularly in different parts of the plant. The spiraling TE bundles reported here and also by Deng et al. (2021) were widespread at the grafting interface of incompatible grafts. We also observed the occurrence of chignon-like sphere structure consisting of small spirals at the *Nb*/*Po* interface (Fig. 2I), which looked like the previously described vascular nodules found in the bud grafting apple tree (Mosse & Labern, 1960). These evidence suggested the formation of the circular vessels might play an important part in grafting process.

Sachs (1984) proposed that the differentiation of circular vessels was related to the circulating flux of auxin. When auxin accumulated near the grafting union, the polarity of signal transmission would be reversed locally with diffusion, resulting in the formation of circular vessels in local areas above the grafting interface. Nevertheless, this explanation might only partially account for the spiraling bundle formation as transverse wounds or injuries usually led to small circular vessels as mentioned above. We did not observe the occurrence of spiraling bundles in the compatible self-grafts of purslane (Table 1), *Arabidopsis* or *Nicotiana* (Table 1; Deng et al., 2021), or of quinoa (Liu et al., 2022). The repeated formation of circular vessel at the grafting interface indicated that the spiraling structure could result from the distant scion-rootstock interaction, or alternatively, simply from the emergent property of perturbed auxin flux by heterografting, or both. In addition, it’s easy to comprehend that the spiraling TE bundles could also occur, though in a low frequency, at the rootstock region below the grafting interface where auxin accumulation was much less due to scion-rootstock disconnection.

## Conclusions

Spiraling TE bundles existed extensively in incompatible grafts. They restricted the graft growth and potentially blocked the vascular connection, thus could be used an important indicator of incompatibility of distant grafts. In practice, we can use this structure to examine the possible grafting relationship between scion and rootstock. Furthermore, future study could be designed to address the origin and molecular mechanisms of spiraling TE bundles.

##  Supplemental Information

10.7717/peerj.14020/supp-1 Supplemental Information 1 Raw data for Fig. 2B, E, J, K

## Additional Information and Declarations

### Data Availability

The following information was supplied regarding data availability: The raw data is available in the Supplemental File.
